# The reverse Warburg effect is likely to be an Achilles' heel of cancer that can be exploited for cancer therapy

**DOI:** 10.18632/oncotarget.18175

**Published:** 2017-05-25

**Authors:** Yaojie Fu, Shanshan Liu, Shanghelin Yin, Weihong Niu, Wei Xiong, Ming Tan, Guiyuan Li, Ming Zhou

**Affiliations:** ^1^ The Key Laboratory of Carcinogenesis of The Chinese Ministry of Health, Xiangya Hospital, Central South University, Changsha, Hunan 410078, P. R. China; ^2^ Cancer Research Institute, Central South University, Changsha, Hunan 410078, P. R. China; ^3^ Medical School of Xiangya, Central South University, Changsha, Hunan 410013, P. R. China; ^4^ Mitchell Cancer Institute, University of South Alabama, Mobile, AL 36604, USA

**Keywords:** the Warburg effect, the reverse Warburg effect, cancer glucose metabolism, lactate shuttle, oxidative stress

## Abstract

Although survival outcomes of cancer patients have been improved dramatically via conventional chemotherapy and targeted therapy over the last decades, there are still some tough clinical challenges that badly needs to be overcome, such as anticancer drug resistance, inevitable recurrences, cancer progression and metastasis. Simultaneously, accumulated evidence demonstrates that aberrant glucose metabolism termed ‘the Warburg effect’ in cancer cell is closely associated with malignant phenotypes. In 2009, a novel ‘two-compartment metabolic coupling’ model, also named ‘the reverse Warburg effect’, was proposed and attracted lots of attention. Based on this new model, we consider whether this new viewpoint can be exploited for improving the existent anti-cancer therapeutic strategies. Our review focuses on the paradigm shift from ‘the Warburg effect’ to ‘the reverse Warburg effect’, the features and molecular mechanisms of ‘the reverse Warburg effect’, and then we discuss its significance in fundamental researches and clinical practice.

## INTRODUCTION

**Figure 1:**
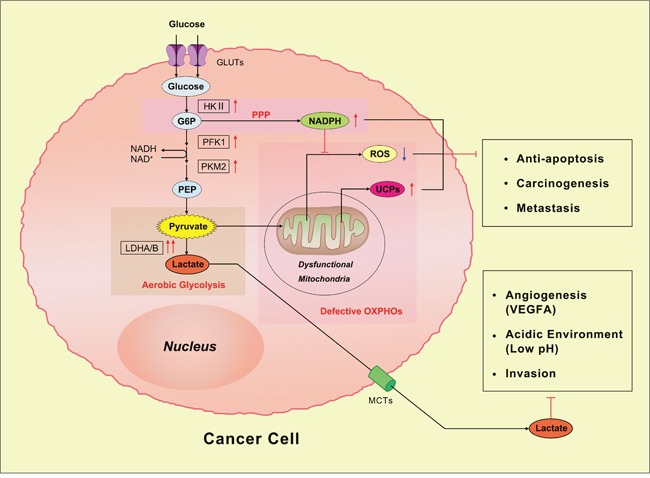
The Warburg effect in cancer cells As shown in this diagram, the Warburg effect is mainly induced by mitochondrial dysfunction. NADPH: nicotinamide adenine dinucleotide phosphate; ROS: reactive oxygen species; UCPs: uncoupling proteins; PEP: phospho-enolpyruvate; GLUTs: glucose transporters; HK: Hexokinase; G6P: glucose 6 phosphate; MCTs: monocarboxylate transporters; PPP: pentose phosphate pathway; PFK1: phosphofructokinase-1; LDHA/B: lactate dehydrogenase A/B.

The production, transformation and utilization of energy in a living organism are essential for various biological activities. Emergences of aberrant metabolic characteristics imply a new pattern for survival and fitness or abnormal biological functions. As one of the several hallmarks of cancer [1], the anomalous characteristic of energy metabolism pathway in cancer cells has received striking attention in the past decades [2–4], which is regarded as important as other features, such as sustained angiogenesis, avoiding immune destruction and so forth [5]. In the 1920s, the metabolic distinction between normal and tumor cells was firstly reported by Otto Warburg [6], termed as ‘the Warburg effect’, which suggests even in the presence of sufficient oxygen, the malignant cells prefer to produce adenosine triphosphate (ATP) via glycolysis Instead of oxidative phosphorylations (OXPHOs) [7]. The process is also called ‘aerobic glycolysis’. As a milestone in cancer research, this remarkable discovery has become a basic principle of energy metabolism for cancer cells in the last several decades. According to ‘the Warburg effect’, the partial suppression of oxidative metabolism in tumor cells is mainly resulted from the mitochondrial dysfunction. It has long been recognized that glycolytic phenotypes in cancer cells are closely connected with defective mitochondrial OXPHOs (Figure 1). For examples, glycolytic enzymes such as lactate dehydrogenase (LDH), phosphofructokinase (PFK) and hexokinaseII(HKII) increase under hypoxic condition, which has been associated with anti-apoptosis and even ‘chemoresistance’ [8, 9]. The overproduced LDHA and LDHB catalyze the conversion of pyruvate into lactate, consequently, lactate is secreted into microenvironment through monocarboxylate transporters (MCTs), which contributes to extracellular acidity [10]. The local acidified environment can facilitate cancer invasion via an increase in vascular endothelial growth factor A (VEGFA) [11, 12]. Simultaneously, the elevated level of NADPH through pentose phosphate pathway (PPP) serves as byproducts of proliferation or cofactor for supplement of reduced glutathione (GSH) [13]. The excess NADPH not only induces the reduction of reactive oxygen species (ROS), it's also closely linked to anti-apoptosis and initiation of metastasis under oxidative stress. The decrease of manganese superoxide dismutase (MnSOD), an important mitochondrially localized enzyme, is responsible for adaptive increase of uncoupling proteins (UCPs) and then leads to carcinogenesis and glycolytic metabolism diagram [14, 15]. Compared to OXPHOs, ‘the Warburg effect’ contributes to anti-apoptosis, increasing biosynthesis of macromolecules and balancing the intercellular redox potential [2, 4]. In addition, this original work undoubtedly provides a new avenue to oncologic research and clinical medicine, such as PET/CT, an effective diagnostic method that is widely used in many fields [16, 17]. However, increasing evidence indicates limitations and questionable points of ‘the Warburg effect’. The theory of ‘aerobic glycolysis’ was challenged by some current investigations, for example, in many human malignant cell lines, glycolysis contributes less than 50% for energy production [18]. Especially in cervical and breast cancer cell lines, such as HeLa and MCFs, mitochondrial OXPHOs still contributes 79% and 91% respectively in overall ATP generation under normal conditions, it only reduces to less than 40% in hypoxic environment [9, 19, 20]. And stunningly, Suganuma et al reported that leukemia THP-1 may be a newly identified ‘oxidative’ cell line, which is sensitive to OXPHOs inhibitor oligomycin A, inversely, it is resistant to glycolytic inhibitor 2-DG [21]. More importantly, the Warburg effect merely focuses on metabolism in cancer cell and neglected the metabolic interactions between cancer cells and other components in microenvironment [22].

### Metabolic interaction between aerobic and oxidative cells indicates a paradigm shift to the reverse Warburg effect

Unlike the Warburg effect, some tumor cells exhibit high rates of OXPHOs [13, 14, 23]. In these cells, glycolysis contributes 1% to 64% of ATP production, OXPHOs is still the predominant ATP supplier for cancer cells [24, 25]. Moreover, many studies show that OXPHOs and aerobic glycolysis are not always mutually exclusive, to some extent, they contribute differently to ATP production with the alterations in the tumor environment, such as in normoxia and in hypoxia [26, 27]. It implies that ‘the Warburg effect’ is not a general feature of all cancers, heterogeneous tumor cells exhibit flexible metabolic phenotypes even in a single tumor mass [28–31]. Furthermore, the most intriguing question is still the ‘metabolic paradox’: how to explain that ATP is produced via a low efficiency method despite high energy demand for tumor proliferation and metastasis?

The revealed studies pointed out that OXPHOs was not constantly suppressed during carcinogenesis, it could be partly restored under nutrient shortage status via metabolic reprogramming induced by LKB1-AMPK-p53 and PI3K-Akt-mTOR pathways [32]. Clinically, 3-bromophyruvate (3-BP), a potent glycolysis inhibitor has been proved not always helpful in cancer patients [33]. Recent clinical material shows that in breast cancer the mitochondrial respiration significantly increases and is sensitive to respiratory chain inhibitors [31, 34, 35]. Thus, we need to reconsider why seemingly peripheral mitochondrial OXPHOs in ‘the Warburg effect’ greatly affect clinical the outcomes of cancer patients.

**Figure 2:**
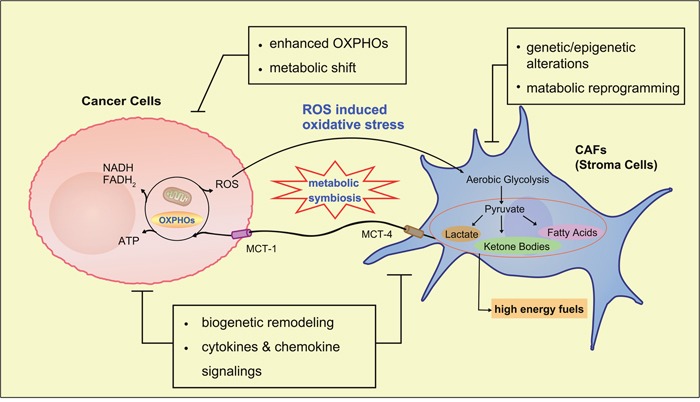
The reverse Warburg effect Cancer cells induce *oxidative stress* in neighboring fibroblasts by secreting reactive oxygen species (*ROS*), triggering aerobic glycolysis and production of high energy metabolites, especially lactate and pyruvate, which are in turn transported through ‘*lactate shuttle*’ to sustain the anabolic need of adjacent cancer cells. In this process, many events occur such as loss of Cav-1 in stroma cells, upregulation of mono-carboxylate transporters (MCTs) in both, etc. These changes mean more than biomarkers of increased aerobic glycolysis in stroma cells, but are involved in some regulatory pathways which drive tumor progression, metastasis and even drug resistance.

Recently, more experiments indicate that tumor-microenviroment (TME) plays a key role in carcinogenesis and epithelial-mesenchymal transition (EMT) [36, 37]. Stromal cells constituting dominantly in the microenvironment, especially the cancer-associated fibroblasts (CAFs), affect the homeostasis of TME. Futhermore, interactions between cancer cells and surrounding CAFs highly affect the growth, metabolism, metastasis and progression of carcinoma [31, 38]. Based on this renewed interest, a ‘two-compartment’ model, also named as ‘the reverse Warburg effect’, has been proposed to reconsider metabolism in tumor [39–42]. It has rapidly attracted considerable attention. In this model, cancer cells and CAFs become metabolically coupled (Figure 2). Cancer cells secrete hydrogen peroxide into microenvironment, which induces oxidative stress in neighboring CAFs. Consequently, CAFs undergo aerobic glycolysis and generate high level of energy-rich fuels (such as pyruvate, ketone bodies, fatty acids and lactate), in turn, these energy-rich fuels ‘feed’ mitochondrial OXPHOs in cancer cells and are utilized for efficient ATP production [43–46]. In this pattern, loss of Caveolin-1 (Cav-1) in stromal cells, an important structural protein that is involved in several regulations including signaling pathways, endocytosis and vesicular transport, may exacerbate oxidative stress and mitochondrial dysfunctions in CAFs [43, 47–49]. In addition, upregulated mono-carboxylate transporters (MCTs) serve as an ‘energy transfer device’, which mediates transportation of high energy fuels (such as lactate) from CAFs to cancer cells. It has been termed ‘lactate shuttle’ [31, 44, 45, 50–54].

### Unraveled mechanisms involved in the reverse Warburg effect

#### Oxidative stress induced metabolic interaction

Oxidative stress is undoubtedly core part in metabolic reprogramming of two-compartment model of ‘the reverse Warburg effect’ [44, 55, 56]. It plays a critical role as a ‘mutagenic’ and ‘metabolic’ engine driving DNA damage, abnormal chromosome numbers (aneuploidy) and CAFs-cancer cell co-evolution [57, 58]. In addition, gene profiling implies that oxidative stress also affects expression of some molecules and then regulates autophagy, mitophagy, inflammatory responses, apoptosis and other behaviors in CAFs [58–60].

First of all, oxidative stress related loss of stromal caveolin-1 (Cav-1) seems one of the most important predictive biomarkers of tumor recurrence, invasion and prognosis [49, 61]. In the theory of ‘the autophagic tumor stroma model of cancer metabolism’, cancer cells secrete ROS in microenvironment, which induces oxidative stress in CAFs, and then leads to the onset of autophagy and production of auto-phagosomes that fuse with lysosomes, resulting in degradation of mitochondria and Caveolin-1 (Cav-1). In turn, loss of Cav-1 causes more production of ROS in cancer cells, which initiates the cascade of oxidative stress in CAFs through a positive feedback mechanism [44, 58, 62, 63]. Moreover, apart from oxidative stress, there are other possible mechanisms that may participate in deregulation of Cav-1 in CAFs, such as activation of TGF-beta signaling pathway, inactivation of tumor suppress genes (such as p53) and activation of oncogenes (H-ras, v-abl, brc-abl, TGF etc.) [64, 65]. As a potent inhibitor of nitric oxide synthase (NOS), Cav-1 also binds to it and inhibits its activity in stroma cells. Therefore, CAFs are unable to restrict nitric oxide (NO) synthesis due to loss of Cav-1, and accumulation of NO induces mitochondrial dysfunction and glycolytic metabolism [47, 66]. In general, different pathways modulate suppression of Cav-1 and ROS production, which contribute to metabolic shift from mitochondrial OXPHOS to glycolysis in CAFs [67].

Secondly, oxidative stress elevates two main transcription factors: hypoxia inducible factor 1α (HIF-1α) and nuclear factor κB (NFκB) [68–70]. Some studies revealed that activation of HIF-1α and NFκB is mainly mediated by reduced expression of prolyl hydroxylase domain–containing protein (PHD) [71]. Under normal physiological oxygen concentration, α-subunit of HIF is hydroxylated by PHD and subsequently degraded by E3-ligase and Hippel-Lindau (VHL) protein [72]. However, accumulated ROS or hypoxic environment prevents production of PHD, reduces hydroxylation level of HIF-1α and activates it. More details about the procedure are not so clear yet [73]. As for NFκB, it is activated by IκB kinase (IκBK), which is under the control of oxygen-sensitive PHD. So downregulation of PHD induces activation of IκBK, consequently, mediates activation of NFκB [74, 75]. As a regulator of innate immune response, it also effects secretion of cytokines and metabolic pattern in CAFs [61, 76]. HIF-1α induces hypoxia response, promotes transcription of angiogenic factors (such as VEGF) and mediates autophagy, mitophagy and aerobic glycolysis [77, 78]. In the process of glycolysis, several glycolytic enzymes, such as 2 isoforms of pyruvate kinase M (PKM1 and PKM2), mono-carboxylate transporters (MCTs) and lactate dehydrogenase A and B (LDHA, LDHB) overexpress significantly [79–81]. As the rate limiting glycolytic enzymes, increased expression of PKM1 and PKM2 promote cancer mass growth by different mechanisms. It has been shown that PKM1 upregulation, as a consequence of increased aerobic glycolysis, mediates lactate production in CAFs. Differently, PKM2 upregulation promotes autophagic program in CAFs, and also stimulates ketone bodies storage [82–84]. Recent *in vivo* studies indicate that excess PKM1 increases tumor related inflammation, while overexpressed PKM2 enhances mitochondrial OXPHOS in cancer cells [85, 86]. High level of LDHs and MCTs accelerate energy-rich fuels production and transportation to cancer cells for survival, progression and invasion [56, 87, 88].

**Figure 3:**
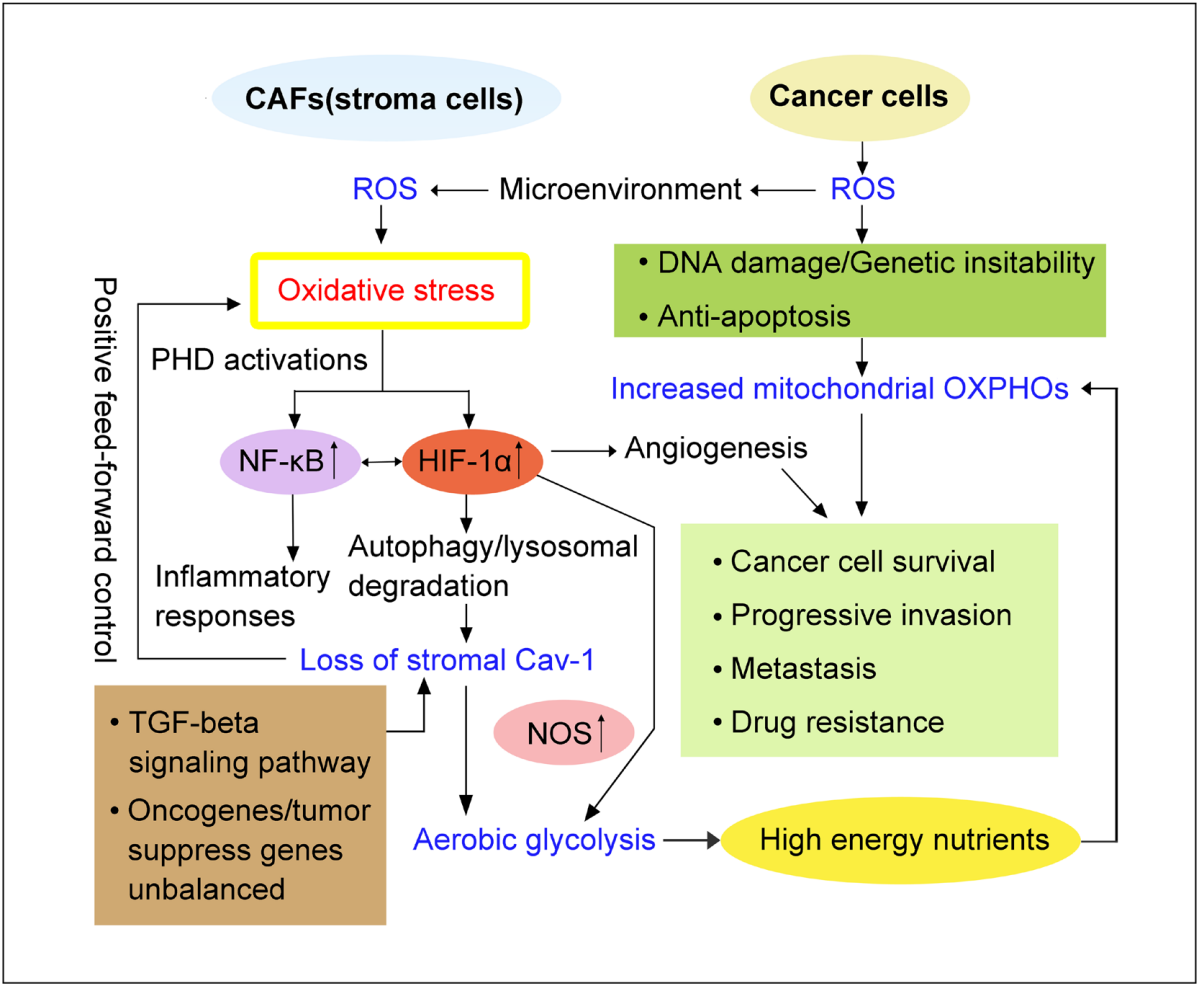
Oxidative stress-mechanism in the reverse Warburg effect Reactive oxygen species (ROS) generated in cancer cells freely diffuses into microenvironment and enters into adjacent CAFs, which results in oxidative stress. Consequently, oxidative stress leads to activation of HIF-1α and NFκB. HIF-1α triggers angiogenesis, aerobic glycolysis. In addition, HIF-1α induces autophagy and lysosomal degradation, which causes loss of stromal Cav-1. As an important structural protein and nitric oxide synthase (NOS) inhibitor, loss of Cav-1 amplifies oxidative stress by ‘positive feed-forward control’ and also contributes to aerobic glycolysis in CAFs. As consequence, glycolytic enzymes such as pyruvate kinase M 1and 2 (PKM1, PKM2), lactate dehydrogenase A and B (LDHA, LDHB), mono-carboxylate transporters (MCTs) are highly activated. Briefly, oxidative stress-mechanism is strongly correlated to the reverse Warburg effect.

Taken together, ‘oxidative stress mechanism’ drives CAFs-Cancer cells metabolic coupling and support tumor growth through genetic, metabolic and cellular processes (Figure 3).

#### Cellular electromagnetic field theory

The reverse Warburg effect is far more than metabolic disturbances in cancer cells or CAFs, which involves insights into structural changes of molecules, biochemical reactions and genetic modifications [89–91]. Some remarkable biophysical evidences provide new perspectives to cancer research. In earlier time, it suggested that coherent electrical polar oscillations and formation of electromagnetic fields play a critical part in living cells, and their disturbances occur in cancer cells, but the exact structure generating electromagnetic field was not identified. Subsequently, extensive researches and series of experimental measurements confirmed that microtubule is the main source of electromagnetic reactions [92–95]. Interestingly, microtubules exhibit considerable influences on mitochondrial activity and affect many biologic activities of living cells, especially in cancer cells. Normal mitochondrial functions mainly depend on transfer of protons from the matrix into the intermembrane space and finally diffuse through outer membrane to cytoplasm. This proton-transfer route is correlated with formation of a strong static electric field and high level of water ordering in the mitochondrial neighborhood. And at the same time, microtubule oscillations, a strongly nonlinear and low damping activity, are activated by energy generated in mitochondria [95]. Importantly, microtubule oscillations are connected with generation of electrodynamic field which involves transport of molecules and particles, and information transfer [96].

In cancer tissue with the reverse Warburg effect, mitochondrial function in CAFs is suppressed by genetic and chemical signaling. Defects in respiratory enzymes and electron carries in inner membrane disturb normal proton transfer. As consequence, it leads to mitochondrial dysfunction and changes of mitochondrial inner membrane potential. In contrary, cancer cells obtain the enhanced mitochondrial activity and uptake high energy rich metabolites (pyruvate, lactate etc.), provided by CAFs and stroma. It provides the increased power of microtubule oscillations, causing proliferation, aggressiveness and metastatic ability [97].

### The relationship between Warburg effect and the reverse Warburg effect: competitive or cooperative?

Based on multi-genetic/epigenetic reprogramming and heterogeneity of different sub-populations of cancer cells, apparently, the Warburg effect can't meet the demands of malignant transformation and development in tumor. As a metabolic symbiosis, the reverse Warburg effect emphasizes metabolic alterations in CAFs and reciprocal interactions with cancer cells. Despite huge differences between the two metabolic phenotypes, they seem to be reconciled with each other due to the complex metabolic plasticity and regulation. Moreover, the metabolic diversity of cancer tissue reflects rapidly adaptation to the drastic changes in nutrient environment [98].

According to the perspective of intra-tumor heterogeneity (ITH), even in a single tumor, there are numerous heterogeneous populations, OXPHOs and glycolysis contribute differently to each population, they both favour tumor tissue metabolism under different conditions. For those oxidative cancer cells, they generate energy in mitochondria at a high rate and metabolically interact with CAFs. Metabolic reprogramming in CAFs, resulting from the direct contact with cancer cells and paracrine signaling in TME, contributes to emergence of the reverse Warburg effect [99]. But for glycolytic cancer cells, the Warburg effect is still predominant.

The ‘competition’ between glycolysis (the Warburg effect) and OXPHOs (the reverse Warburg effect) is mainly influenced by growth requirement. Some studies suggested that for rapid proliferation tumor, glycolysis may be more privileged. Because, apart from plenty energy supply, cancer cells need lipids, nucleic acids and other intermediates from glycolysis for biosynthesis. For differentiated tumors, enhanced OXPHOs is prior for efficient ATP production, which inhibits key enzymes of glycolysis (e.g. phosphofructokinase 1 and pyruvate kinase 1) [100].

In addition, even though the distinction of the Warburg effect and the reverse Warburg effect is emphasized, we have to admit the ‘cooperation’ between them is really important for the dynamic metabolic regulations during carcinogenesis. Smolkova et al put forward that initially the proliferation and differentiation of cancer stem cells (CSCs) are manipulated by oncogenetic (e.g. Myc) signaling. Meanwhile, activation of hypoxia-inducible factor 1 (HIF1), AMPK and NFκB signaling mediates hypoxic conditions, which leads to oncogenic and hypoxia-mediated glycolytic phenotype in cancer cells [32]. Once entering proliferation phase, mitochondrial revival starts in cancer cells with increased consumption of nutrients and oxygen, undergoing the co-evolution with cancer cells, CAFs get genetic/epigenetic reprogramming that induces metabolic alterations and emergence of the reverse Warburg effect [61].

In general, classic Warburg effect and the reverse Warburg effect are not totally competitive, the two metabolic models can be perfectly reconciled with each other as well. It reflects the highly plasticity and dynamicity of cancer glucose metabolism. So it's easier to understand why glycolytic pattern is not the unique method for cancer glucose metabolism, and therapeutics targeting glycolysis in cancer patients not always has positive responses. Considering the metabolic diversity and pattern shift between the co-existing modes: glycolysis and OXPHOs, we speculate whether the reverse Warburg effect may provide new avenues for cancer treatment?

### Targeting ‘the reverse Warburg effect’ may provide a novel strategy for cancer diagnosis and treatment

Greek mythology breeds the earliest misty impression of our fate, and also provides some romantic elements for scientific research. One of the mythological stories says, when Achilles was born, his mother was informed that her son would die young. To prevent this tragedy, she took Achilles to River Styx, dipped his body into the river, which was supposed to offer power of invulnerability. Unfortunately, his heel failed to be washed by the magical river water for his mother held him by that. So, Achilles’ heel is a weakness spot of an overall strong body, which eventually led to his death after shot by a poisonous arrow in the war. Now, from the metabolic perspective in cancer research, we quote this meaningful term from Greek mythology, use it to describe ‘the reverse Warburg effect’ for cancer treatment by analogy with the ‘Achilles heel’ for an unconquerable hero. First, like what we have mentioned above, loss of stromal Cav-1 occurs in various epithelial cancers, especially in nearly all sub-types of human breast cancers. Independent of other epithelial markers (such as HER2, ER, PR), it's a newly identified predicative biomarker of early recurrence, lymph node metastasis, tamoxifen-resistance and poor clinical outcome [48, 101–103]. Moreover, xenograft model experiments indicate that Cav-1 deficient stroma cells drive angiogenesis and tumor growth [104–106]. We have discussed loss of Cav-1 is one of significant reasons which is responsible for aerobic glycolysis in CAFs, correspondingly, it has been shown that combination of two potent mitochondrial ‘poisons’ (metformin and arsenic trioxide (ATO)) are able to re-sensitize breast cancer cells that are CAF-induced tamoxifen and fulvestrant resistance [107]. Similarly, based on ‘the reverse Warburg effect’, for prostate cancer (PCa), many cases show a high expressions of MCT4 and CAIX(an established hypoxia marker) in CAFs with concomitant strong MCT1 expression in PCa cells, which are always connected with high aggressiveness [108, 109]. It suggests treatment with MCT1/MCT4 targeted drugs maybe a good option for improving the poor diagnosis [110]. Recently, metformin, a classic antidiabetic agent for treating type 2 diabetes, has been newly considered for reducing the expression of MCT4 on CAFs. Now, it's undergoing phase 2/3 clinical trials as adjuvant therapy in several cancer types [111]. Moreover, combination of Acetylcysteine (N-Acetyl-L-cysteine) and Topotecan is on phase 2 clinical trial in patients with ovarian cancer, based on their regulation of Cav-1, MCT4 and HIF-1α expression [53].

**Figure 4:**
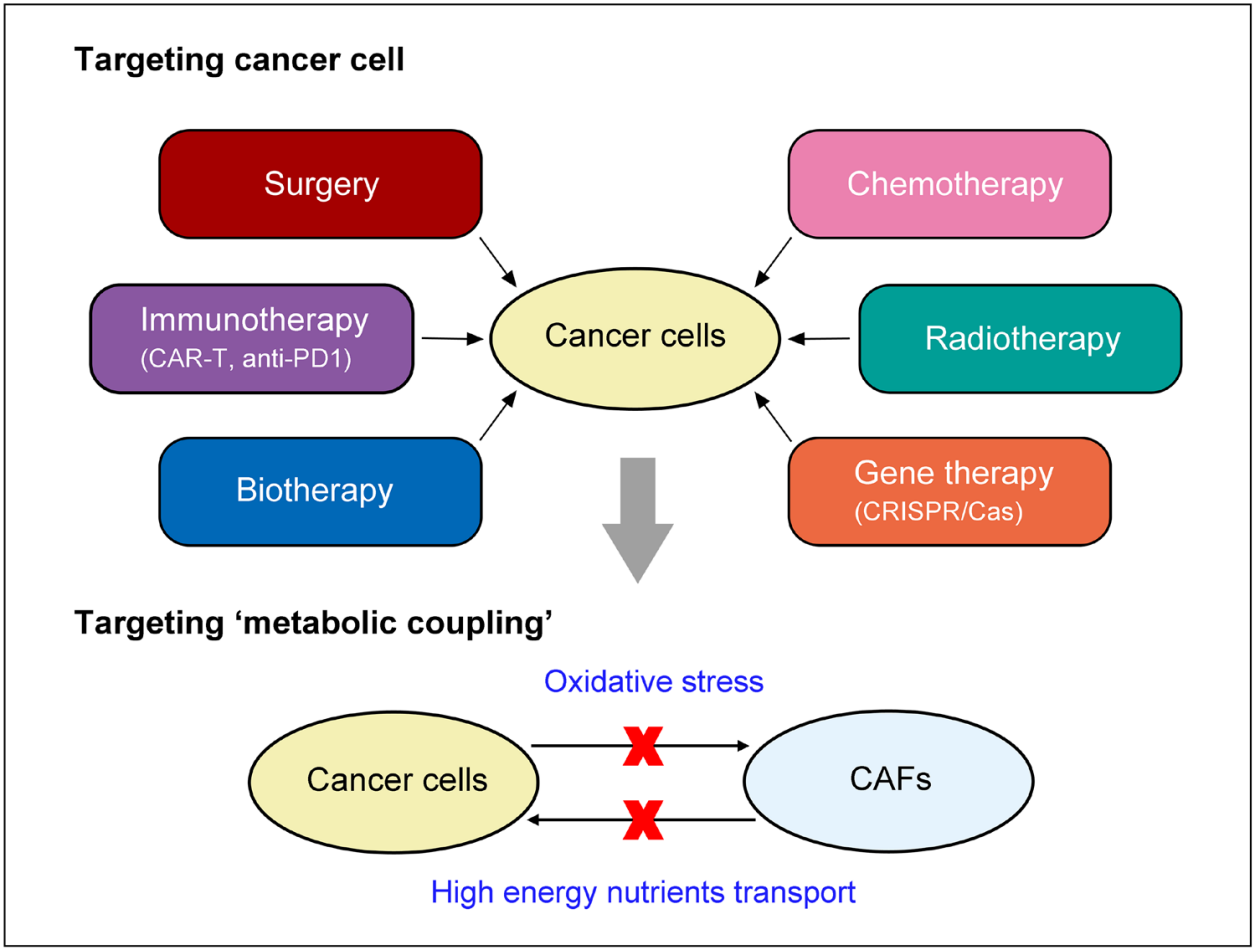
‘Two-compartment blockade’ therapeutic mode based on the reverse Warburg effect This mode targets metabolic interactions between cancer cells and CAFs, including:(1) ROS generated in cancer cells and subsequent oxidative stress on CAFs. (2) High energy metabolites transport from CAFs to cancer cells.

In some patients with aggressive B-cell lymphoma accompanied by lactate acidosis, preclinical studies show that FDA-approved drugs, such as metformin and sirolimus can inhibit activation of ‘lactate shuttle’ (or high expression of MCTs), decrease expression of LDHB and PKM1, suppress CAFs-cancer cells metabolic coupling [112]. Likewise, for prostate cancer patients, there are great differences on clinical courses between Gleason scores (GS) 3 + 3 and 3 + 4 prostate cancers (PCa). It has been confirmed that some reverse Warburg effect related genes (such as FOXO1, GPD2, SPARC, HK2, COLIA2 etc.) are differentially expressed between GP3 and GP4 PCa. Hence, gene expression profiling based on ‘the reverse Warburg effect’ has been used to classify Gleason pattern, distinguishing GP3 from GP4 PCa [113]. Based on ‘the reverse Warburg effect’, we consider whether interfering with the interplay between CAFs and cancer cells is more effective than targeting cancer cells alone? Apart from the existent surgery, radiotherapy, chemotherapeutic drugs, or even some newly emergent cancer treatment, such as the revolutionary use of CRISPR/Cas 9 clinically, delivery of engineered cytotoxic T cells (CAR-T) targeting tumor antigens [114], clinical use of antibodies targeting immune checkpoint blockade like cytotoxic T-lymphocyte associated antigen-4 (CTLA-4) and programmed death-1 (PD-1) [115], we boldly speculate a new therapeutic mode and describe it as ‘two-compartment blockade’. In this mode, two routes maybe promising targeting candidates (Figure 4): (1) ROS generated in cancer cells and subsequent oxidative stress on CAFs. (2) High energy metabolites transport from CAFs to cancer cells.

Despite some applications of the reverse Warburg effect in cancer patients outcome evaluation, early stage diagnosis, even theoretically clinical benefits, we still need further studies to exploit more therapeutic potential of the reverse Warburg effect in the future.

## CONCLUSION

Taken together, the reverse Warburg effect is a novel metabolic pattern newly identified between cancer cells and neighboring Cancer-associated fibroblasts (CAFs). Its discovery doesn't deny the value of the Warburg effect and cannot replace it, actually, the reverse Warburg effect extends the heterogeneity and plasticity of cancer metabolism. It is closely connected with proliferation, metastasis, angiogenesis, drug resistance and other aggressive behaviors of cancer cells. Although it's validated that ‘the reverse Warburg effect’ can be initiated by oxidative stress in two-compartment metabolic coupling and change of cellular electromagnetic field, detailed mechanisms are still not clear. Current findings about the reverse Warburg effect provide series of new predicative biomarkers for cancer and novel strategies for anti-cancer therapies. We believe that this ‘Achilles’ Heel’ will bring new approaches for cancer treatment in the future.

## Abbreviations

NO: nitric oxide
LDH: lactate dehydrogenase
PFK: phosphofructokinase
Cav-1: Caveolin-1
HKII: hexokinaseII
PPP: pentose phosphate pathway
GS: Gleason scores
CAFs: cancer-associated fibroblasts
OXPHOs: oxidative phosphorylations
PHD: prolyl hydroxylase domain–containing protein
EMT: epithelial-mesenchymal transition
PCa: prostate cancers
NOS: nitric oxide synthase
VHL: Hippel-Lindau
TME: tumor-microenviroment
3-BP: 3-bromophyruvate
UCPs: uncoupling proteins
NADPH: nicotinamide adenine dinucleotide phosphate
PKM: pyruvate kinase M
ROS: reactive oxygen species
MCTs: mono-carboxylate transporters
IκBK: IκB kinase
CTLA-4: cytotoxic T-lymphocyte associated antigen-4
PD-1: programmed death-1
NFκB: nuclear factor κB
HIF-1α: hypoxia inducible factor 1α
MnSOD: manganese superoxide dismutase
LDHA/B: lactate dehydrogenase A and B
VEGFA: vascular endothelial growth factor A

## References

[1] Hanahan D, Weinberg RA (2011). Hallmarks of cancer: the next generation. Cell.

[2] Li W, Li X, Wang W, Yi M, Zhou Y, Zheng P, Xiong W, Yang J, Peng S, McCarthy JB, Xiang B, Li G (2013). Tumor suppressor gene Oxidored-nitro domain-containing protein 1 regulates nasopharyngeal cancer cell autophagy, metabolism, and apoptosis in vitro. Int J Biochem Cell Biol.

[3] DeBerardinis RJ, Lum JJ, Hatzivassiliou G, Thompson CB (2008). The biology of cancer: metabolic reprogramming fuels cell growth and proliferation. Cell Metab.

[4] Hsu PP, Sabatini DM (2008). Cancer cell metabolism: Warburg and beyond. Cell.

[5] Muz B, de la Puente P, Azab F, Azab AK (2015). The role of hypoxia in cancer progression, angiogenesis, metastasis, and resistance to therapy. Hypoxia (Auckl).

[6] Warburg O, Wind F, Negelein E (1927). The metabolism of tumors in the body. J Gen Physiol.

[7] Marin D, Sabater B (2017). The cancer Warburg effect may be a testable example of the minimum entropy production rate principle. Phys Biol.

[8] Kalyanaraman B (2017). Teaching the basics of cancer metabolism: developing antitumor strategies by exploiting the differences between normal and cancer cell metabolism. Redox Biol.

[9] Li H, Li X, Ge X, Jia L, Zhang Z, Fang R, Yang J, Liu J, Peng S, Zhou M, Xiang J, Zeng Z, Zhou W (2016). MiR-34b-3 and miR-449a inhibit malignant progression of nasopharyngeal carcinoma by targeting lactate dehydrogenase. A. Oncotarget.

[10] Hay N (2016). Reprogramming glucose metabolism in cancer: can it be exploited for cancer therapy?. Nat Rev Cancer.

[11] Doherty JR, Cleveland JL (2013). Targeting lactate metabolism for cancer therapeutics. J Clin Invest.

[12] Shi Q, Le X, Wang B, Abbruzzese JL, Xiong Q, He Y, Xie K (2001). Regulation of vascular endothelial growth factor expression by acidosis in human cancer cells. Oncogene.

[13] Jiang P, Du W, Wu M (2014). Regulation of the pentose phosphate pathway in cancer. Protein Cell.

[14] Kamarajugadda S, Cai Q, Chen H, Nayak S, Zhu J, He M, Jin Y, Zhang Y, Ai L, Martin SS, Tan M, Lu J (2013). Manganese superoxide dismutase promotes anoikis resistance and tumor metastasis. Cell Death Dis.

[15] Xu Y, Miriyala S, Fang F, Bakthavatchalu V, Noel T, Schell DM, Wang C, St Clair WH, St Clair DK (2015). Manganese superoxide dismutase deficiency triggers mitochondrial uncoupling and the Warburg effect. Oncogene.

[16] Anastasiou D, Poulogiannis G, Asara JM, Boxer MB, Jiang JK, Shen M, Bellinger G, Sasaki AT, Locasale JW, Auld DS, Thomas CJ, Vander HM, Cantley LC (2011). Inhibition of pyruvate kinase M2 by reactive oxygen species contributes to cellular antioxidant responses. Science.

[17] Cairns RA, Harris IS, Mak TW (2011). Regulation of cancer cell metabolism. Nat Rev Cancer.

[18] Zu XL, Guppy M (2004). Cancer metabolism: facts, fantasy, and fiction. Biochem Biophys Res Commun.

[19] Hernandez-Resendiz I, Roman-Rosales A, Garcia-Villa E, Lopez-Macay A, Pineda E, Saavedra E, Gallardo-Perez JC, Alvarez-Rios E, Gariglio P, Moreno-Sanchez R, Rodriguez-Enriquez S (2015). Dual regulation of energy metabolism by p53 in human cervix and breast cancer cells. Biochim Biophys Acta.

[20] Rodriguez-Enriquez S, Carreno-Fuentes L, Gallardo-Perez JC, Saavedra E, Quezada H, Vega A, Marin-Hernandez A, Olin-Sandoval V, Torres-Marquez ME, Moreno-Sanchez R (2010). Oxidative phosphorylation is impaired by prolonged hypoxia in breast and possibly in cervix carcinoma. Int J Biochem Cell Biol.

[21] Suganuma K, Miwa H, Imai N, Shikami M, Gotou M, Goto M, Mizuno S, Takahashi M, Yamamoto H, Hiramatsu A, Wakabayashi M, Watarai M, Hanamura I (2010). Energy metabolism of leukemia cells: glycolysis versus oxidative phosphorylation. Leuk Lymphoma.

[22] Das R, Strowig T, Verma R, Koduru S, Hafemann A, Hopf S, Kocoglu MH, Borsotti C, Zhang L, Branagan A, Eynon E, Manz MG, Flavell RA (2016). Microenvironment-dependent growth of preneoplastic and malignant plasma cells in humanized mice. Nat Med.

[23] Mandujano-Tinoco EA, Gallardo-Perez JC, Marin-Hernandez A, Moreno-Sanchez R, Rodriguez-Enriquez S (2013). Anti-mitochondrial therapy in human breast cancer multi-cellular spheroids. Biochim Biophys Acta.

[24] Zheng J (2012). Energy metabolism of cancer: glycolysis versus oxidative phosphorylation (review). Oncol Lett.

[25] Fan TW, Kucia M, Jankowski K, Higashi RM, Ratajczak J, Ratajczak MZ, Lane AN (2008). Rhabdomyosarcoma cells show an energy producing anabolic metabolic phenotype compared with primary myocytes. Mol Cancer.

[26] Alfarouk KO, Shayoub ME, Muddathir AK, Elhassan GO, Bashir AH (2011). Evolution of tumor metabolism might reflect carcinogenesis as a reverse evolution process (dismantling of multicellularity). Cancers (Basel).

[27] Xu XD, Shao SX, Jiang HP, Cao YW, Wang YH, Yang XC, Wang YL, Wang XS, Niu HT (2015). Warburg effect or reverse Warburg effect? A review of cancer metabolism. Oncol Res Treat.

[28] Lee M, Yoon JH (2015). Metabolic interplay between glycolysis and mitochondrial oxidation: the reverse Warburg effect and its therapeutic implication. World J Biol Chem.

[29] Gatenby RA, Smallbone K, Maini PK, Rose F, Averill J, Nagle RB, Worrall L, Gillies RJ (2007). Cellular adaptations to hypoxia and acidosis during somatic evolution of breast cancer. Br J Cancer.

[30] Ertel A, Tsirigos A, Whitaker-Menezes D, Birbe RC, Pavlides S, Martinez-Outschoorn UE, Pestell RG, Howell A, Sotgia F, Lisanti MP (2012). Is cancer a metabolic rebellion against host aging? In the quest for immortality, tumor cells try to save themselves by boosting mitochondrial metabolism. Cell Cycle.

[31] Migneco G, Whitaker-Menezes D, Chiavarina B, Castello-Cros R, Pavlides S, Pestell RG, Fatatis A, Flomenberg N, Tsirigos A, Howell A, Martinez-Outschoorn UE, Sotgia F, Lisanti MP (2010). Glycolytic cancer associated fibroblasts promote breast cancer tumor growth, without a measurable increase in angiogenesis: evidence for stromal-epithelial metabolic coupling. Cell Cycle.

[32] Smolkova K, Plecita-Hlavata L, Bellance N, Benard G, Rossignol R, Jezek P (2011). Waves of gene regulation suppress and then restore oxidative phosphorylation in cancer cells. Int J Biochem Cell Biol.

[33] Xu RH, Pelicano H, Zhou Y, Carew JS, Feng L, Bhalla KN, Keating MJ, Huang P (2005). Inhibition of glycolysis in cancer cells: a novel strategy to overcome drug resistance associated with mitochondrial respiratory defect and hypoxia. Cancer Res.

[34] Quarato G, Piccoli C, Scrima R, Capitanio N (2011). Variation of flux control coefficient of cytochrome c oxidase and of the other respiratory chain complexes at different values of protonmotive force occurs by a threshold mechanism. Biochim Biophys Acta.

[35] Kaambre T, Chekulayev V, Shevchuk I, Karu-Varikmaa M, Timohhina N, Tepp K, Bogovskaja J, Kutner R, Valvere V, Saks V (2012). Metabolic control analysis of cellular respiration in situ in intraoperational samples of human breast cancer. J Bioenerg Biomembr.

[36] Neelakantan D, Drasin DJ, Ford HL (2015). Intratumoral heterogeneity: clonal cooperation in epithelial-to-mesenchymal transition and metastasis. Cell Adh Migr.

[37] Junk DJ, Cipriano R, Bryson BL, Gilmore HL, Jackson MW (2013). Tumor microenvironmental signaling elicits epithelial-mesenchymal plasticity through cooperation with transforming genetic events. Neoplasia.

[38] Orimo A, Gupta PB, Sgroi DC, Arenzana-Seisdedos F, Delaunay T, Naeem R, Carey VJ, Richardson AL, Weinberg RA (2005). Stromal fibroblasts present in invasive human breast carcinomas promote tumor growth and angiogenesis through elevated SDF-1/CXCL12 secretion. Cell.

[39] Yoshida GJ (2015). Metabolic reprogramming: the emerging concept and associated therapeutic strategies. J Exp Clin Cancer Res.

[40] Sotgia F, Whitaker-Menezes D, Martinez-Outschoorn UE, Flomenberg N, Birbe RC, Witkiewicz AK, Howell A, Philp NJ, Pestell RG, Lisanti MP (2012). Mitochondrial metabolism in cancer metastasis: visualizing tumor cell mitochondria and the “reverse Warburg effect” in positive lymph node tissue. Cell Cycle.

[41] Saada A (2014). Mitochondria: mitochondrial OXPHOS (dys) function ex vivo--the use of primary fibroblasts. Int J Biochem Cell Biol.

[42] Pavlides S, Whitaker-Menezes D, Castello-Cros R, Flomenberg N, Witkiewicz AK, Frank PG, Casimiro MC, Wang C, Fortina P, Addya S, Pestell RG, Martinez-Outschoorn UE, Sotgia F (2009). The reverse Warburg effect: aerobic glycolysis in cancer associated fibroblasts and the tumor stroma. Cell Cycle.

[43] Arcucci A, Ruocco MR, Granato G, Sacco AM, Montagnani S (2016). Cancer: an oxidative crosstalk between solid tumor cells and cancer associated fibroblasts. Biomed Res Int.

[44] Bonuccelli G, Whitaker-Menezes D, Castello-Cros R, Pavlides S, Pestell RG, Fatatis A, Witkiewicz AK, Vander HM, Migneco G, Chiavarina B, Frank PG, Capozza F, Flomenberg N (2010). The reverse Warburg effect: glycolysis inhibitors prevent the tumor promoting effects of caveolin-1 deficient cancer associated fibroblasts. Cell Cycle.

[45] Pertega-Gomes N, Vizcaino JR, Attig J, Jurmeister S, Lopes C, Baltazar F (2014). A lactate shuttle system between tumour and stromal cells is associated with poor prognosis in prostate cancer. BMC Cancer.

[46] Galina A (2014). Mitochondria: 3-bromopyruvate vs. mitochondria? A small molecule that attacks tumors by targeting their bioenergetic diversity. Int J Biochem Cell Biol.

[47] Witkiewicz AK, Dasgupta A, Nguyen KH, Liu C, Kovatich AJ, Schwartz GF, Pestell RG, Sotgia F, Rui H, Lisanti MP (2009). Stromal caveolin-1 levels predict early DCIS progression to invasive breast cancer. Cancer Biol Ther.

[48] Witkiewicz AK, Dasgupta A, Sammons S, Er O, Potoczek MB, Guiles F, Sotgia F, Brody JR, Mitchell EP, Lisanti MP (2010). Loss of stromal caveolin-1 expression predicts poor clinical outcome in triple negative and basal-like breast cancers. Cancer Biol Ther.

[49] Sotgia F, Del GF, Casimiro MC, Bonuccelli G, Mercier I, Whitaker-Menezes D, Daumer KM, Zhou J, Wang C, Katiyar S, Xu H, Bosco E, Quong AA (2009). Caveolin-1−/− null mammary stromal fibroblasts share characteristics with human breast cancer-associated fibroblasts. Am J Pathol.

[50] Whitaker-Menezes D, Martinez-Outschoorn UE, Lin Z, Ertel A, Flomenberg N, Witkiewicz AK, Birbe RC, Howell A, Pavlides S, Gandara R, Pestell RG, Sotgia F, Philp NJ (2011). Evidence for a stromal-epithelial “lactate shuttle” in human tumors: MCT4 is a marker of oxidative stress in cancer-associated fibroblasts. Cell Cycle.

[51] Witkiewicz AK, Whitaker-Menezes D, Dasgupta A, Philp NJ, Lin Z, Gandara R, Sneddon S, Martinez-Outschoorn UE, Sotgia F, Lisanti MP (2012). Using the “reverse Warburg effect” to identify high-risk breast cancer patients: stromal MCT4 predicts poor clinical outcome in triple-negative breast cancers. Cell Cycle.

[52] Rae C, Nasrallah FA, Broer S (2009). Metabolic effects of blocking lactate transport in brain cortical tissue slices using an inhibitor specific to MCT1 and MCT2. Neurochem Res.

[53] Cirri P, Chiarugi P (2012). Cancer-associated-fibroblasts and tumour cells: a diabolic liaison driving cancer progression. Cancer Metastasis Rev.

[54] Choi J, Kim DH, Jung WH, Koo JS (2013). Metabolic interaction between cancer cells and stromal cells according to breast cancer molecular subtpe. Breast Cancer Res.

[55] Costa A, Scholer-Dahirel A, Mechta-Grigoriou F (2014). The role of reactive oxygen species and metabolism on cancer cells and their microenvironment. Semin Cancer Biol.

[56] Martinez-Outschoorn UE, Balliet RM, Rivadeneira DB, Chiavarina B, Pavlides S, Wang C, Whitaker-Menezes D, Daumer KM, Lin Z, Witkiewicz AK, Flomenberg N, Howell A, Pestell RG (2010). Oxidative stress in cancer associated fibroblasts drives tumor-stroma co-evolution: a new paradigm for understanding tumor metabolism, the field effect and genomic instability in cancer cells. Cell Cycle.

[57] Fiaschi T, Chiarugi P (2012). Oxidative stress, tumor microenvironment, and metabolic reprogramming: a diabolic liaison. Int J Cell Biol.

[58] Martinez-Outschoorn UE, Lisanti MP, Sotgia F (2014). Catabolic cancer-associated fibroblasts transfer energy and biomass to anabolic cancer cells, fueling tumor growth. Semin Cancer Biol.

[59] Lisanti MP, Martinez-Outschoorn UE, Sotgia F (2013). Oncogenes induce the cancer-associated fibroblast phenotype: metabolic symbiosis and “fibroblast addiction” are new therapeutic targets for drug discovery. Cell Cycle.

[60] Fordyce CA, Patten KT, Fessenden TB, DeFilippis R, Hwang ES, Zhao J, Tlsty TD (2012). Cell-extrinsic consequences of epithelial stress: activation of protumorigenic tissue phenotypes. Breast Cancer Res.

[61] Martinez-Outschoorn UE, Curry JM, Ko YH, Lin Z, Tuluc M, Cognetti D, Birbe RC, Pribitkin E, Bombonati A, Pestell RG, Howell A, Sotgia F, Lisanti MP (2013). Oncogenes and inflammation rewire host energy metabolism in the tumor microenvironment: RAS and NFkappaB target stromal MCT4. Cell Cycle.

[62] Palozza P, Sestito R, Picci N, Lanza P, Monego G, Ranelletti FO (2008). The sensitivity to beta-carotene growth-inhibitory and proapoptotic effects is regulated by caveolin-1 expression in human colon and prostate cancer cells. Carcinogenesis.

[63] Chen D, Che G (2014). Value of caveolin-1 in cancer progression and prognosis: emphasis on cancer-associated fibroblasts, human cancer cells and mechanism of caveolin-1 expression (Review). Oncol Lett.

[64] Bist A, Fielding CJ, Fielding PE (2000). p53 regulates caveolin gene transcription, cell cholesterol, and growth by a novel mechanism. Biochemistry.

[65] Guido C, Whitaker-Menezes D, Capparelli C, Balliet R, Lin Z, Pestell RG, Howell A, Aquila S, Ando S, Martinez-Outschoorn U, Sotgia F, Lisanti MP (2012). Metabolic reprogramming of cancer-associated fibroblasts by TGF-beta drives tumor growth: connecting TGF-beta signaling with “Warburg-like” cancer metabolism and L-lactate production. Cell Cycle.

[66] Pavlides S, Tsirigos A, Migneco G, Whitaker-Menezes D, Chiavarina B, Flomenberg N, Frank PG, Casimiro MC, Wang C, Pestell RG, Martinez-Outschoorn UE, Howell A, Sotgia F (2010). The autophagic tumor stroma model of cancer: role of oxidative stress and ketone production in fueling tumor cell metabolism. Cell Cycle.

[67] Sotgia F, Martinez-Outschoorn UE, Pavlides S, Howell A, Pestell RG, Lisanti MP (2011). Understanding the Warburg effect and the prognostic value of stromal caveolin-1 as a marker of a lethal tumor microenvironment. Breast Cancer Res.

[68] Martinez-Outschoorn UE, Trimmer C, Lin Z, Whitaker-Menezes D, Chiavarina B, Zhou J, Wang C, Pavlides S, Martinez-Cantarin MP, Capozza F, Witkiewicz AK, Flomenberg N, Howell A (2010). Autophagy in cancer associated fibroblasts promotes tumor cell survival: role of hypoxia, HIF1 induction and NFkappaB activation in the tumor stromal microenvironment. Cell Cycle.

[69] Chiavarina B, Whitaker-Menezes D, Migneco G, Martinez-Outschoorn UE, Pavlides S, Howell A, Tanowitz HB, Casimiro MC, Wang C, Pestell RG, Grieshaber P, Caro J, Sotgia F (2010). HIF1-alpha functions as a tumor promoter in cancer associated fibroblasts, and as a tumor suppressor in breast cancer cells: autophagy drives compartment-specific oncogenesis. Cell Cycle.

[70] Capparelli C, Whitaker-Menezes D, Guido C, Balliet R, Pestell TG, Howell A, Sneddon S, Pestell RG, Martinez-Outschoorn U, Lisanti MP, Sotgia F (2012). CTGF drives autophagy, glycolysis and senescence in cancer-associated fibroblasts via HIF1 activation, metabolically promoting tumor growth. Cell Cycle.

[71] Semenza GL (2010). HIF-1: upstream and downstream of cancer metabolism. Curr Opin Genet Dev.

[72] Panday A, Inda ME, Bagam P, Sahoo MK, Osorio D, Batra S (2016). Transcription factor NF-kappaB: an update on intervention strategies. Arch Immunol Ther Exp (Warsz).

[73] Zwaans BM, Lombard DB (2014). Interplay between sirtuins, MYC and hypoxia-inducible factor in cancer-associated metabolic reprogramming. Dis Model Mech.

[74] Pantuck AJ, An J, Liu H, Rettig MB (2010). NF-kappaB-dependent plasticity of the epithelial to mesenchymal transition induced by Von Hippel-Lindau inactivation in renal cell carcinomas. Cancer Res.

[75] Zhao R, Liu Y, Wang H, Yang J, Niu W, Fan S, Xiong W, Ma J, Li X, Phillips JB, Tan M, Qiu Y, Li G (2016). BRD7 plays an anti-inflammatory role during early acute inflammation by inhibiting activation of the NF-κB signaling pathway. Cell Mol Immunol.

[76] Ren Q, Kari C, Quadros MR, Burd R, McCue P, Dicker AP, Rodeck U (2006). Malignant transformation of immortalized HaCaT keratinocytes through deregulated nuclear factor kappaB signaling. Cancer Res.

[77] De Francesco EM, Lappano R, Santolla MF, Marsico S, Caruso A, Maggiolini M (2013). HIF-1alpha/GPER signaling mediates the expression of VEGF induced by hypoxia in breast cancer associated fibroblasts (CAFs). Breast Cancer Res.

[78] Zhou F, Du J, Wang J (2017). Albendazole inhibits HIF-1alpha-dependent glycolysis and VEGF expression in non-small cell lung cancer cells. Mol Cell Biochem.

[79] Pavlides S, Tsirigos A, Vera I, Flomenberg N, Frank PG, Casimiro MC, Wang C, Pestell RG, Martinez-Outschoorn UE, Howell A, Sotgia F, Lisanti MP (2010). Transcriptional evidence for the “Reverse Warburg Effect” in human breast cancer tumor stroma and metastasis: similarities with oxidative stress, inflammation, Alzheimer's disease, and “Neuron-Glia Metabolic Coupling”. Aging (Albany NY).

[80] Chiavarina B, Whitaker-Menezes D, Martinez-Outschoorn UE, Witkiewicz AK, Birbe R, Howell A, Pestell RG, Smith J, Daniel R, Sotgia F, Lisanti MP (2011). Pyruvate kinase expression (PKM1 and PKM2) in cancer-associated fibroblasts drives stromal nutrient production and tumor growth. Cancer Biol Ther.

[81] Giannoni E, Taddei ML, Morandi A, Comito G, Calvani M, Bianchini F, Richichi B, Raugei G, Wong N, Tang D, Chiarugi P (2015). Targeting stromal-induced pyruvate kinase M2 nuclear translocation impairs oxphos and prostate cancer metastatic spread. Oncotarget.

[82] Li Z, Yang P, Li Z (2014). The multifaceted regulation and functions of PKM2 in tumor progression. Biochim Biophys Acta.

[83] Hamabe A, Konno M, Tanuma N, Shima H, Tsunekuni K, Kawamoto K, Nishida N, Koseki J, Mimori K, Gotoh N, Yamamoto H, Doki Y, Mori M (2014). Role of pyruvate kinase M2 in transcriptional regulation leading to epithelial-mesenchymal transition. Proc Natl Acad Sci U S A.

[84] Chaneton B, Gottlieb E (2012). Rocking cell metabolism: revised functions of the key glycolytic regulator PKM2 in cancer. Trends Biochem Sci.

[85] Yang W, Lu Z (2013). Regulation and function of pyruvate kinase M2 in cancer. Cancer Lett.

[86] Tommelein J, Verset L, Boterberg T, Demetter P, Bracke M, De Wever O (2015). Cancer-associated fibroblasts connect metastasis-promoting communication in colorectal cancer. Front Oncol.

[87] Rattigan YI, Patel BB, Ackerstaff E, Sukenick G, Koutcher JA, Glod JW, Banerjee D (2012). Lactate is a mediator of metabolic cooperation between stromal carcinoma associated fibroblasts and glycolytic tumor cells in the tumor microenvironment. Exp Cell Res.

[88] Qiao A, Gu F, Guo X, Zhang X, Fu L (2016). Breast cancer-associated fibroblasts: their roles in tumor initiation, progression and clinical applications. Front Med.

[89] Quail DF, Joyce JA (2013). Microenvironmental regulation of tumor progression and metastasis. Nat Med.

[90] Hanahan D, Coussens LM (2012). Accessories to the crime: functions of cells recruited to the tumor microenvironment. Cancer Cell.

[91] Destefanis M, Viano M, Leo C, Gervino G, Ponzetto A, Silvagno F (2015). Extremely low frequency electromagnetic fields affect proliferation and mitochondrial activity of human cancer cell lines. Int J Radiat Biol.

[92] Patruno A, Amerio P, Pesce M, Vianale G, Di Luzio S, Tulli A, Franceschelli S, Grilli A, Muraro R, Reale M (2010). Extremely low frequency electromagnetic fields modulate expression of inducible nitric oxide synthase, endothelial nitric oxide synthase and cyclooxygenase-2 in the human keratinocyte cell line HaCat: potential therapeutic effects in wound healing. Br J Dermatol.

[93] Pokorny J, Vedruccio C, Cifra M, Kucera O (2011). Cancer physics: diagnostics based on damped cellular elastoelectrical vibrations in microtubules. Eur Biophys J.

[94] Bellorofonte C, Vedruccio C, Tombolini P, Ruoppolo M, Tubaro A (2005). Non-invasive detection of prostate cancer by electromagnetic interaction. Eur Urol.

[95] Bonnet S, Archer SL, Allalunis-Turner J, Haromy A, Beaulieu C, Thompson R, Lee CT, Lopaschuk GD, Puttagunta L, Bonnet S, Harry G, Hashimoto K, Porter CJ (2007). A mitochondria-K+ channel axis is suppressed in cancer and its normalization promotes apoptosis and inhibits cancer growth. Cancer Cell.

[96] Pokorny J, Martan T, Foletti A (2012). High capacity optical channels for bioinformation transfer: acupuncture meridians. J Acupunct Meridian Stud.

[97] Pokorny J, Pokorny J, Kobilkova J (2013). Postulates on electromagnetic activity in biological systems and cancer. Integr Biol (Camb).

[98] Penkert J, Ripperger T, Schieck M, Schlegelberger B, Steinemann D, Illig T (2016). On metabolic reprogramming and tumor biology: a comprehensive survey of metabolism in breast cancer. Oncotarget.

[99] Fiaschi T, Marini A, Giannoni E, Taddei ML, Gandellini P, De Donatis A, Lanciotti M, Serni S, Cirri P, Chiarugi P (2012). Reciprocal metabolic reprogramming through lactate shuttle coordinately influences tumor-stroma interplay. Cancer Res.

[100] Lunt SY, Vander HM (2011). Aerobic glycolysis: meeting the metabolic requirements of cell proliferation. Annu Rev Cell Dev Biol.

[101] El-Gendi SM, Mostafa MF, El-Gendi AM (2012). Stromal caveolin-1 expression in breast carcinoma. Correlation with early tumor recurrence and clinical outcome. Pathol Oncol Res.

[102] Shan-Wei W, Kan-Lun X, Shu-Qin R, Li-Li Z, Li-Rong C (2012). Overexpression of caveolin-1 in cancer-associated fibroblasts predicts good outcome in breast cancer. Breast Care (Basel).

[103] Martinez-Outschoorn UE, Pavlides S, Whitaker-Menezes D, Daumer KM, Milliman JN, Chiavarina B, Migneco G, Witkiewicz AK, Martinez-Cantarin MP, Flomenberg N, Howell A, Pestell RG, Lisanti MP (2010). Tumor cells induce the cancer associated fibroblast phenotype via caveolin-1 degradation: implications for breast cancer and DCIS therapy with autophagy inhibitors. Cell Cycle.

[104] Di Vizio D, Morello M, Sotgia F, Pestell RG, Freeman MR, Lisanti MP (2009). An absence of stromal caveolin-1 is associated with advanced prostate cancer, metastatic disease and epithelial Akt activation. Cell Cycle.

[105] Eliyatkin N, Aktas S, Diniz G, Ozgur HH, Ekin ZY, Kupelioglu A (2017). Expression of stromal caveolin-1 may be a predictor for aggressive behaviour of breast cancer. Pathol Oncol Res.

[106] Senetta R, Stella G, Pozzi E, Sturli N, Massi D, Cassoni P (2013). Caveolin-1 as a promoter of tumour spreading: when, how, where and why. J Cell Mol Med.

[107] Mao Y, Keller ET, Garfield DH, Shen K, Wang J (2013). Stromal cells in tumor microenvironment and breast cancer. Cancer Metastasis Rev.

[108] Andersen S, Solstad O, Moi L, Donnem T, Eilertsen M, Nordby Y, Ness N, Richardsen E, Busund LT, Bremnes RM (2015). Organized metabolic crime in prostate cancer: the coexpression of MCT1 in tumor and MCT4 in stroma is an independent prognosticator for biochemical failure. Urol Oncol.

[109] Pertega-Gomes N, Vizcaino JR, Miranda-Goncalves V, Pinheiro C, Silva J, Pereira H, Monteiro P, Henrique RM, Reis RM, Lopes C, Baltazar F (2011). Monocarboxylate transporter 4 (MCT4) and CD147 overexpression is associated with poor prognosis in prostate cancer. BMC Cancer.

[110] Gurrapu S, Jonnalagadda SK, Alam MA, Nelson GL, Sneve MG, Drewes LR, Mereddy VR (2015). Monocarboxylate transporter 1 inhibitors as potential anticancer agents. ACS Med Chem Lett.

[111] Romero IL, Mukherjee A, Kenny HA, Litchfield LM, Lengyel E (2015). Molecular pathways: trafficking of metabolic resources in the tumor microenvironment. Clin Cancer Res.

[112] Martinez-Outschoorn UE, Whitaker-Menezes D, Valsecchi M, Martinez-Cantarin MP, Dulau-Florea A, Gong J, Howell A, Flomenberg N, Pestell RG, Wagner J, Arana-Yi C, Sharma M, Sotgia F (2013). Reverse Warburg effect in a patient with aggressive B-cell lymphoma: is lactic acidosis a paraneoplastic syndrome?. Semin Oncol.

[113] Georgescu I, Gooding RJ, Doiron RC, Day A, Selvarajah S, Davidson C, Berman DM, Park PC (2016). Molecular characterization of Gleason patterns 3 and 4 prostate cancer using reverse Warburg effect-associated genes. Cancer Metab.

[114] Boice M, Salloum D, Mourcin F, Sanghvi V, Amin R, Oricchio E, Jiang M, Mottok A, Denis-Lagache N, Ciriello G, Tam W, Teruya-Feldstein J, de Stanchina E (2016). Loss of the HVEM tumor suppressor in lymphoma and restoration by modified CAR-T cells. Cell.

[115] Topalian SL, Hodi FS, Brahmer JR, Gettinger SN, Smith DC, McDermott DF, Powderly JD, Carvajal RD, Sosman JA, Atkins MB, Leming PD, Spigel DR, Antonia SJ (2012). Safety, activity, and immune correlates of anti-PD-1 antibody in cancer. N Engl J Med.

